# Default mode network-basal ganglia network connectivity predicts the transition to postherpetic neuralgia^☆^

**DOI:** 10.1016/j.ibneur.2025.01.009

**Published:** 2025-01-13

**Authors:** Ying Wu, Chao Wang, Wei Qian, Lieju Wang, Lina Yu, Minming Zhang, Min Yan

**Affiliations:** a Department of Anesthesiology, the Second Affiliated Hospital, Zhejiang University School of Medicine, Hangzhou 310000, China; b Department of Radiology, the Second Affiliated Hospital, Zhejiang University School of Medicine, Hangzhou 310000, China

**Keywords:** Postherpetic neuralgia, Acute herpes zoster, FMRI, Network functional connectivity, Predict outcome

## Abstract

**Background:**

Neuroimaging studies have revealed aberrant network functional connectivity in postherpetic neuralgia (PHN) patients. However, there is a lack of knowledge regarding the relationship between the brain network connectivity during the acute period and disease prognosis.

**Objective:**

The purpose of this study was to detect characteristic network connectivity in the process of herpes zoster (HZ) pain chronification and to identify whether abnormal network connectivity in the acute period can predict the outcome of patients with HZ.

**Methods:**

In this cross-sectional study, 31 patients with PHN, 33 with recuperation from herpes zoster (RHZ), and 28 with acute herpes zoster (AHZ) were recruited and underwent resting-state functional magnetic resonance imaging (fMRI). We investigated the differences in the connectivity of four resting-state networks (RSN) among the three groups. Receiver operating characteristic (ROC) curve analysis was performed to identify whether abnormal network connectivity in the acute period could predict the outcome of patients with HZ.

**Results:**

First, we found within-basal ganglia network (BGN) and default mode network (DMN)-BGN connectivity differences, with PHN patients showing increased DMN-BGN connectivity compared to AHZ and RHZ patients, while RHZ patients showing increased within-BGN connectivity compared to AHZ and PHN patients. Moreover, DMN-BGN connectivity was associated with the ID pain score in patients with AHZ. Finally, the DMN-BGN connectivity of AHZ patients could predict the outcome of HZ patients with sensitivity and specificity of 77.8 % and 63.2 %, respectively.

**Conclusions:**

Our results provide evidence that DMN-BGN connectivity during the acute period confers a risk for the development of chronic pain and can act as a neuroimaging biomarker to predict the outcome of patients with HZ.

## 1 Introduction

Herpes zoster (HZ) is characterized by acute skin inflammation that presents as shingles and severe pain caused by peripheral nerve injury. Postherpetic neuralgia (PHN) is the most intractable complication of Herpes zoster (HZ) (Johnson and Rice, 2014). Epidemiological results indicate that 12.5 % of HZ patients aged ≥ 50 years develop PHN 3 months after the rash, and that the proportion affected increases markedly with advancing age (Forbes et al., 2016). These patients experience persistent pain and emotional and cognitive distress for months or even years, which dramatically degrades quality of life for a large sector of society (Johnson et al., 2010, Weaver, 2007). However, it remains unclear why some patients with similarly painful shingle episodes recover, while others remain affected by persistent pain. It is important to determine whether factors other than patient age and the initial severity of acute pain and rash contribute to the risk of pain chronification in HZ (Forbes et al., 2016). Previous human and animal studies have shown that chronic pain is associated with peripheral and central nervous system reorganization, encompassing a long list of neuronal and glial changes associated with pain persistence (Baron et al., 2010). However, owing to the lack of an entirely adequate animal model to mimic the PHN disease course, our understanding of the central mechanism and risk of HZ chronification based on animal experiments is hampered. Thus, a clinical neuroimaging approach may help reveal brain changes during the course of HZ.

Neuroimaging studies have revealed aberrant brain structure and function in regions that are emotion/reward-linked (such as the amygdala and striatum) in PHN patients, in addition to sensory-discriminative regions, compared with healthy subjects or acute HZ patients (Geha et al., 2007, Geha et al., 2008, Hong et al., 2018, Cao et al., 2017, Zhang et al., 2014, Gu et al., 2019, Zhang et al., 2016). In addition, another longitudinal study showed that PHN brains exhibit abnormal regional homogeneity (ReHo), fractional aptitude of low-frequency fluctuation (fALFF), and voxel-based morphometry (VBM) values in the pain matrix (the frontal lobe, thalamus, limbic lobe, and cerebellum), occipital lobe, and temporal lobe compared to HZ brains (Cao et al., 2018). These results suggest that brain functional and structural changes may be correlated with HZ-PHN chronification.

However, chronic pain, a higher-grade brain function, relies on the collaboration of large-scale networks, rather than on single regions in isolation. Our previous study found that a brain-network-level imbalance involving the default mode network (DMN), salience network (SN), emotion regulation network (ERN), and basal ganglia network (BGN) could account for pain-related dysfunction in different outcomes of HZ patients (Wu et al., 2022). However, the distinctive network connectivity of acute herpes zoster (AHZ) is important to understand the mechanism of occurrence and development in HZ progress. Thus, in the present study, we brought the AHZ group into the comparisons and continued investigating the differences of network connectivities between different HZ periods, encompassing AHZ, PHN, and recuperation from herpes zoster (RHZ) patients. By adding the acute populations, the aim of present study was to identify the specific network connectivity in the process of HZ chronification.

In addition, brain functional and structural changes not only play a role in the initial development of pain chronification, but can also be used as biomarkers to predict future disease symptoms in several chronic pain conditions, including chronic back pain and urologic chronic pelvic pain syndrome (Baliki et al., 2012, Vachon-Presseau et al., 2016, Mansour et al., 2013, Kutch et al., 2017). Previous studies have not determined the relationship between brain abnormalities in the acute period and the transition to different outcomes in patients with HZ. Accordingly, we explored the relationship between abnormal network connectivity and disease prognosis in patients with AHZ. This may provide a new neuroimaging biomarker that can predict the longitudinal outcome of HZ, permit timely treatment, and reduce the incidence of the PHN transition. If a biological neuroimaging factor predicting the outcome of HZ can be identified, novel treatments directed toward this risk factor may have the potential to promote more sustained symptom abatement in HZ patients.

## 2 Materials and methods

### 2.1 Subjects inclusion and exclusion

In this observational study, 28 AHZ, 31 PHN, and 33 RHZ patients were recruited from the Second Affiliated Hospital of Zhejiang University, School of Medicine, signed informed consent forms in accordance with the approval of the Ethics Committee and followed the consistent recruitment procedures. All the subjects were right-handed and over the age of 50. A pain specialist diagnosed PHN according to the criteria of the International Association for the Study of Pain (IASP), with spontaneous pain intensity of at least 4 on a Visual Analog Scale (VAS) [ranging from 0 (no pain) to 10 (the worst pain imaginable)] and with disease duration ≥ 3 months after the acute trunk or acral shingle episode (Scholz et al., 2019). In contrast, RHZ patients were defined as having a VAS score of < 4, at least 3 months after acute shingles, and no administration of medication. AHZ patients were diagnosed with a VAS score ≥ 4 in conjunction with trunk or acral shingles during the first clinical visit.

For exclusion criteria of all participants, we ruled out any subjects who had the following conditions: (1) atypical HZ (such as eye, ear, visceral HZ, asymptomatic HZ); (2) current ongoing acute or chronic pain conditions, such as arthritis, toothaches, headaches, and lumbar or cervical spondylopathy; (3) any psychiatric or neurological disorder such as epilepsy, history of head injury, or mood disorders; (4) any major severe diseases, such as renal insufficiency or severe cardiovascular diseases with unstable hemodynamics; (5) receipt of any other ongoing medications (with the exception of the medications for PHN treatment); and (6) any MRI contraindications.

### 2.2 Questionnaire assessment

All participants were given a questionnaire assessment 1 h prior to brain scanning, including the short-form McGill Pain Questionnaire (MPQ), VAS (a total score of pain intensity, ranging from 0 to 10), present pain intensity (PPI, the present score of pain intensity before scanning), ID pain score (a score assessing neuropathic pain), Hamilton Depression Scale (HAMD) and Hamilton Anxiety Scale (HAMA), Positive Affect Negative Affect Score (PANAS), and short-form health survey (SF-36) questionnaire (including the following assessment: physical functioning, role-physical, bodily pain, general health, vitality, social functioning, role-emotional, mental health, and reported health transition), similar to our previous study (Wu et al., 2022).

### 2.3 Imaging acquisition

All MRI scans were performed using a 3.0-Tesla MRI scanner (GE Discovery 750) equipped with an 8-channel head coil at the Department of Radiology. During the MRI scan, each patient lay supine with their head firmly restrained using foam pads; earplugs were provided to reduce noise during the scan. High-resolution structural T1-weighted images were acquired using a fast spoiled gradient recalled sequence using the following parameters: repetition time (TR)/echo time (TE) = 7.3/3.0 ms, flip angle = 11°, field of view (FOV) = 260 × 260 mm^2^, matrix size = 256 × 256, slice thickness = 1.2 mm, 196 continuous sagittal slices. Resting-state fMRI images were acquired with a gradient-recalled echo (GRE)-echo planar imaging (EPI) sequence: TR/TE 2000/30 ms, flip angle = 77°, matrix = 64 × 64, FOV = 240 × 240 mm^2^, slice thickness = 4 mm, slice gap = 0 mm, 38 interleaved axial slices, and scan time = 5 min 20 s.

### 2.4 Resting-state fMRI Imaging preprocessing

Data preprocessing was performed using the Resting-State fMRI Data Analysis Toolkit (Rest, V1.8; http://www.restfmri.net) and Statistical Parametric Mapping (SPM12; http://www.fil.ion.ucl.ac.uk/spm) on MATLAB platform (MathWorks, Natick, MA, USA). The imging pre-processing included the following steps: (1) the first 10 volumes of each functional time series were discarded to avoid transient signal changes before the magnetic field reached a steady state and to allow the subjects to adjust to the scanning environment. (2) We corrected the remaining images for timing differences (37th slice as the reference slice). (3)The thresholds for translation (mm) and rotation (degrees) for each participant were obtained by using six parameters (three for translation and three for rotation) for each scan. No slices were discarded in the PHN, RHZ, or AHZ groups because of head motion, considering > 2 mm of displacement or 2° of rotation. (4) Then we performed the spatial normalization to Montreal Neurological Institute (MNI) space using EPI templates with a resampling voxel size of 3 × 3 × 3 mm^3^. (5) Functional data were spatially smoothed using a Gaussian kernel of 6 mm × 6 mm × 6 mm full width at half-maximum (FWHM). (6) Nuisance covariate regression, including head motion parameters, global mean signal, white matter signal, cerebrospinal fluid signal, and other covariates, was removed. (7) Detrending and bandpass temporal filtering (0.01–0.08 Hz) were finally contucted.

### 2.5 Definition of resting-state networks (RSNs) and within- and cross-network functional connectivity (FC) analyses

According to our previous study, four well-established large-scale networks (DMN, SN, ERN, and BGN) with 36 target regions of interest (ROIs) were chosen, which were deemed to actively participate in pain-related sensory, emotional processing and cognitive functions (Wu et al., 2022). FC was quantified using the correlation between the average time series in each ROI and the other ROI time series in the four networks, using Pearson’s correlation coefficient (r). Next, we converted the r values to Z scores by using Fisher’s r-to-Z transformation. Finally, according to a previous study (Nusslock et al., 2019), the Z scores of all internal pairwise connections were averaged to compute the total network value reflecting within-network FC. To assess the strength of the cross-network FC, the Fisher-transformed correlation between each node in one network and the nodes in another network was calculated. We then averaged the Fisher-transformed Pearson correlations of all connections to obtain a single value representing the cross-network connectivity. The FC matrix was used to visualize pairwise correlations between the ROIs of the different RSNs (Wu et al., 2022).

### 2.6 Statistical analysis

Differences in demographic and clinical variables between the PHN, RHZ, and AHZ groups were statistically examined using SPSS 24.0. Data for continuous variables are expressed as mean ± SD and were tested for normality using the Shapiro-Wilk test. Comparisons among groups of variables with no evidence against normality were assessed using an independent one-way ANOVA for significance. Categorical variables were compared among groups using the χ^2^ test or Fisher's exact test. Statistical significance was set at *p*-value < 0.05.

Network FC differences among the three groups were assessed using one-way ANOVA, with age, educational level, and head motion parameters as covariates. All the *p* values of network FC differences were adjusted for multiple comparisons with FDR correction (10 times, comprising 4 within-network comparisons and 6 cross-network comparisons). Post-hoc testing was performed to compare pairwise differences between each pair of groups, the same as our previous study (Wu et al., 2022).

Partial correlation analyses were performed for each group to investigate the correlations between abnormal network FC and clinical characteristics, eliminating the effects of age, years of education, and head-motion parameters. Statistical p values were set < 0.05, with FDR correction (55 times, from 11 clinical measures across 5 network connectivities).

Finally, the outcomes of patients with AHZ were determined via telephone interviews, which served as outcome variables. With the abnormal network connectivity of AHZ patients as the test variable, a receiver operating characteristic (ROC) curve with sensitivity and specificity was shown to quantify the predictive value of PHN prognosis using brain network connectivity in AHZ patients.

## 3 Results

### 3.1 Demographic and clinical characteristics

This study included 92 subjects, including 31 patients with PHN, 33 with RHZ, and 28 with AHZ. Demographic and clinical characteristics of the participants are shown in Table 1. There were statistically significant differences among the PHN, RHZ, and AHZ patients in terms of age (years, mean ± SD: PHN, 65.52 ± 7.78; RHZ, 61.21 ± 6.17; AHZ, 61.50 ± 8.33) and educational level (years, mean ± SD: PHN, 7.56 ± 3.71; RHZ, 8.97 ± 3.41; AHZ, 7.82 ± 4.45). There were no statistically significant differences in gender (male/female: PHN, 21/10; RHZ, 16/20; AHZ, 18/10) among the groups. PHN patients had clinical symptoms similar to those of AHZ patients, including pain and emotional and quality of life (SF-36) scores, with the exception of a lower ID pain score. RHZ patients had lower pain-related and negative emotional scores and a higher quality of life score than PHN and AHZ patients.

**Table 1:** Demographic and clinical variables of postherpetic neuralgia(PHN), recuperation from herpes zoster (RHZ) and acute herpes zoster (AHZ) patients.

	PHN (n = 31)	RHZ (n = 33)	AHZ (n = 28)	F	*p* value
**Demographic data**					
Age (years, mean ± SD)	65.52 ± 7.78	61.21 ± 6.17	61.50 ± 8.33	3.25	0.043*
Gender (males/females)	21/10	16/20	18/10	4.37	0.112
Education (years,mean± SD)	7.56 ± 3.71	8.97 ± 3.41	7.82 ± 4.45	1.21	0.03*
Duration from first onset (months, mean ± SD)	14.92 ± 18.01^a^	8.52 ± 5.53^b^	0.41 ± 0.11	12.89	＜0.001*
**Clinical data**					
Laterality (Left/Right) Rash location Cervical Lumbar Sacral	16/15 4 25 1 1	18/15 6 26 1 0	16/15 4 16 5 3	0.405	0.817
Pain VAS(0−10) (mean ± SD)	5.35 ± 1.02^c^	0.82 ± 0.98^b^	5.67 ± 1.25	199.65	＜0.001*
MPQ sensory (mean ± SD)	7.16 ± 2.7^c^	0.61 ± 0.74^b^	8.1 ± 2.81	103.80	＜0.001*
MPQ affective (mean ± SD)	2.03 ± 1.08^c^	0.12 ± 0.33^b^	2.18 ± 1.52	36.86	＜0.001*
PPI (mean ± SD)	2.52 ± 0.57^c^	0.52 ± 0.51^b^	2.61 ± 0.5	159.78	＜0.001*
ID pain (mean ± SD)	2.42 ± 1.09^a,c^	0.33 ± 0.48^b^	3.36 ± 0.91	101.19	＜0.001*
**Psychological data**					
HAMD (mean ± SD)	6.55 ± 3.09^c^	2.55 ± 2.41^b^	7.00 ± 3.81	19.54	＜0.001*
HAMA (mean ± SD)	4.13 ± 2.42^c^	1.42 ± 1.56^b^	3.93 ± 2.07	17.43	＜0.001*
PANAS positive (mean ± SD)	12.32 ± 1.56^c^	15.52 ± 2.91^b^	12.89 ± 1.95	18.52	＜0.001*
PANAS negative (mean ± SD)	16.16 ± 3.13^c^	10.64 ± 1.32^b^	16.46 ± 3.79	41.16	＜0.001*
SF−36 (mean ± SD)	114.19 ± 7.44^c^	127.94 ± 5.77^b^	110.01 ± 7.56	57.27	＜0.001*
**Head motion parameters**					
Translation(mm,mean ± SD)	0.27 ± 0.14	0.27 ± 0.11	0.28 ± 0.12	0.134	0.85
Translation(mm,mean ± SD)	0.39 ± 0.23	0.38 ± 0.25	0.39 ± 0.22	0.023	0.97

^a^Significant difference between AHZ and PHN

^b^Significant difference between AHZ and RHZ

^c^Significant difference between PHN and RHZ

PHN, postherpetic neuralgia; RHZ, recuperation from herpes zoster;AHZ, acute herpes zoster; VAS, Visual Analogous Scale; MPQ, McGill pain questionnaire; PPI, present pain intensity; HAMD, Hamilton Depression Scale; HAMA, Hamilton Anxiety Scale; PANAS, Positive Affect Negative Affect Score; SF-36, 36-item short form from health survey.

* Significant difference between three groups

### 3.2 Comparison of network FCs among PHN, RHZ, and AHZ patients

**Fig. 1:**
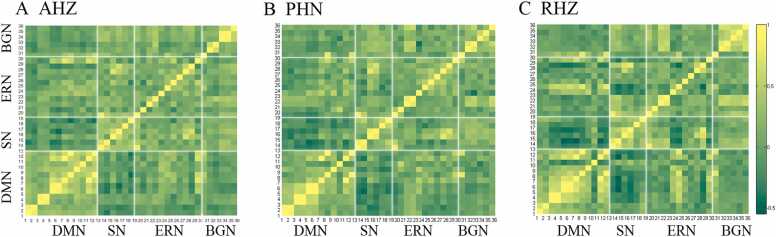
The within- and cross-network functional connectivity matrixes of AHZ(A), PHN(B) and RHZ(C) patients. PHN, postherpetic neuralgia; RHZ, recuperation from herpes zoster; AHZ, acute herpes zoster.

**Fig. 2:**
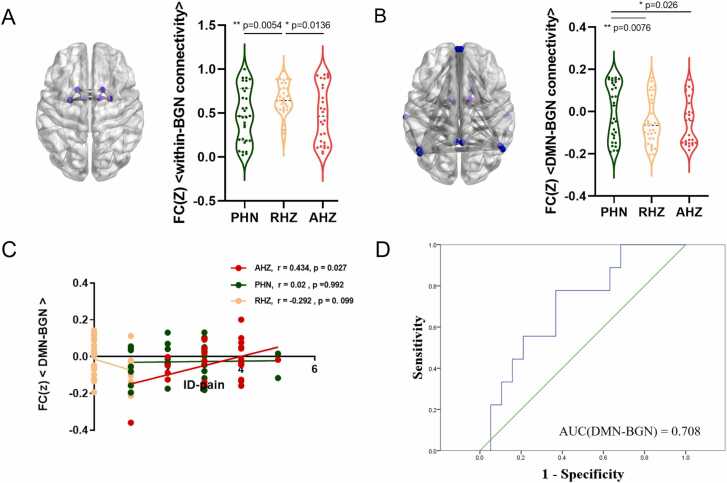
**Comparison of within- and cross networks functional connectivities between PHN, RHZ and AHZ patients**. (A) The significant differences of within-BGN connectivity; (B) The significant differences of DMN-BGN connectivity; (C) Correlation between DMN-BGN connectivity and ID-pain score; (D) Receiver operating characteristic (ROC) curve for predictive value. PHN, postherpetic neuralgia; RHZ, recuperation from herpes zoster; AHZ, acute herpes zoster. DMN, default mode network; BGN, basal ganglia network.

We determined the within- and cross-network FC matrices of AHZ **(**Fig. 1**A)**, PHN **(**Fig. 1**B)**, and RHZ **(**Fig. 1**C)**. One-way ANOVA identified significant abnormal connectivities among patients with PHN, RHZ, and AHZ (FDR-corrected, *p* < 0.05), including within-BGN (*p* = 0.0174) **(**Fig. 2**A)** and DMN-BGN (*p* = 0.0135) connectivity **(**Fig. 2**B)**. There were no significant differences in other network connectivities among the three groups **(**Supplemental Table 1**).**

RHZ patients showed a significant pairwise difference in within-BGN connectivity compared with AHZ (*p* = 0.0136) and PHN (*p* = 0.0054) patients **(**Fig. 2**A)**. PHN patients showed a significant pairwise difference in DMN-BGN connectivity compared with AHZ (*p* = 0.026) and RHZ (*p* = 0.0076) patients **(**Fig. 2**B)**. In short, the DMN-BGN and within-BGN connectivity may provide clues for HZ chronification.

### 3.3 Correlation patterns of network connectivities with clinical symptoms

Partial correlation analysis showed that DMN-BGN connectivity was positively associated with the ID pain score (r = 0.432, *p* = 0.027) in patients with AHZ, with no other significance in the PHN and RHZ groups **(**Fig. 2**C)**. However, this finding did not survive the multiple comparison corrections at an FDR of 5 %. As reported previously (Cao et al., 2020), this correlation could provide a heuristic cue that DMN-BGN connectivity potentially accounts for neuropathic pain in the acute period. No other significant correlations were found between within-BGN connectivity and the clinical variables in patients with AHZ (Supplemental Table 2). No significant correlations were found between other FCs and clinical variables in patients with PHN or RHZ.

### 3.4 Network connectivity predicts pain chronification

Finally, we tracked the outcomes of patients with AHZ through telephonic interviews. An ROC curve was plotted to quantify the predictive value of the HZ prognosis. The DMN-BGN connectivity of AHZ patients predicted the outcome of HZ patients with a sensitivity and specificity of 77.8 % and 63.2 %, respectively. The area under the curve (AUC) was 0.708 (Fig. 2D).

## 4 Discussion

In this study, we provided a relatively comprehensive description of the mechanism of HZ chronification. First, we identified within-BGN and DMN-BGN connectivities by HZ patient outcome, with PHN patients showing increased DMN-BGN connectivity compared to AHZ and RHZ patients and RHZ patients showing increased within-BGN connectivity compared to AHZ and PHN patients. Moreover, the DMN-BGN connectivity was associated with the ID pain score in patients with AHZ and could act as a neuroimaging biomarker to predict HZ patients outcomes.

It is increasingly being recognized that higher-grade brain functions rely on the collaboration of large-scale networks rather than on single regions in isolation (Smith et al., 2013, Fox and Raichle, 2007, Bassett and Sporns, 2017). Researchers have reported the involvement of some RSNs in multiple chronic pain populations and emotional disorders such as chronic orofacial neuropathic pain, temporomandibular disorder, spondyloarthritis, and major depressive disorder (Yu et al., 2019, Kucyi et al., 2014, Hemington et al., 2018, Alshelh et al., 2018). Our previous research also showed that PHN patients could be characterized by abnormal DMN-BGN and within-BGN connectivity compared with RHZ patients (Wu et al., 2022), which is also a key point of the present study. Therefore, we continued to discover the mechanism of HZ pain chronification from the perspective of the RSNs connectivity.

The principal anatomical component of BGN is the striatum, which is divided into caudate, putamen, and nucleus accumbens (NAc). The BGN receives afferent inputs from different cortical regions and projects to the cortex via the thalamus, forming a thalamocorticostriatal loop (Borsook et al., 2010, Luscher and Malenka, 2011). This neuronal loop serves as the basis of pain processing which encompasses the sensory-discriminative, emotional/affective, and cognitive dimensions of pain (Borsook et al., 2010, Chudler and Dong, 1995, Cropley et al., 2006). In particular, the pivotal role of the NAc, a central node of the mesocorticolimbic system, in reward aversion processing makes it a key component of the emotional pain network (Baliki et al., 2013). The striatum of the BGN (especially NAc) is rich in dopaminergic neurons and is considered a hedonic node involving reward, addiction, and euphoric mood (Serafini et al., 2020). Studies have demonstrated that prominent connectivity between the NAc and dorsal striatum (caudate/putamen) is related to emotional learning (Chang et al., 2014, Voorn et al., 2004). NAc structural and functional abnormalities exist in pain and depression independently and within comorbid states (Serafini et al., 2020, Sheng et al., 2017, Pittenger and Duman, 2008). The strength of within-BGN connectivity may be one of the central mechanisms involved in pain-depression comorbidity. Our finding of a higher strength of within-BGN connectivity and positive affective scores in RHZ patients suggests that positive emotions may contribute to the process of pain recovery, which can be partly accounted by within-BGN dopaminergic neurons. In contrast, the lower-level within-BGN connectivity and higher negative emotional scores in patients with AHZ and PHN suggest that persistent low-level within-BGN connectivity may be involved in the pain-depression vicious circle and may promote pain development and maintenance. Unfortunately, the correlation between within-BGN connectivity and clinical scores did not survive, mainly because the pain and emotional scores in the same group were over-centralized and could not widen the gap, while the times of correlation analysis were too many to achieve a significant value. However, as reported previously (Cao et al., 2020), a similar tendency of within-BGN connectivity and affective score may provide a heuristic cue that within-BGN connectivity potentially accounts for pain alleviation in the RHZ group.

Another network emphasized in our results is the DMN, which is involved in internally directed processes, including theory of mind and memory, attention, and mind-wandering (Peer et al., 2015, Raichle, 2015, Spreng and Grady, 2010). Previous studies have shown that DMN dynamics are disrupted in several chronic pain diseases (Kucyi et al., 2013, Baliki et al., 2008), such as migraine (Xue et al., 2012), fibromyalgia (Napadow et al., 2010), complex regional pain syndrome (Bolwerk et al., 2013), and chronic back pain (Loggia et al., 2013). The imbalanced DMN pattern in these diseases is likely associated with pain attention, rumination, and pain-related negative emotion (Kucyi et al., 2014, Kucyi et al., 2013, Baliki et al., 2008, Erpelding and Davis, 2013). In addition, abnormal DMN activity has been linked to difficulty in regulating negative thinking in depressive disorders (Malhi et al., 2019, Malhi et al., 2018, Rogers and Joiner, 2017), indirectly suggesting that the DMN is involved in the integration of pain and depression. In general, these findings indicate that chronic pain does indeed have a widespread effect on DMN balance and suggest that DMN disruptions may underlie the emotional and cognitive impairments accompanying chronic pain (Baliki et al., 2008). Furthermore, several human and animal studies have identified the role of the medial prefrontal cortex-NAc (part of DMN-BGN) circuitry in chronic pain persistence and its emotional regulation, which has also been emphasized in our study (Vachon-Presseau et al., 2016, Chang et al., 2014, Ren et al., 2016, Lee et al., 2015, Zhou et al., 2018). Our results showed higher DMN-BGN connectivity in PHN patients than AHZ patients, suggesting that frequent information exchange between emotion-related BGN and advanced cognition-related DMN during the disease course of PHN plays an important role in pain chronification, probably partly by aggravating the degree of pain rumination and pain-related negative mood. Our study is consistent with a longitudinal study showing variation in mPFC-NAc connectivity in a population with chronic back pain population (Baliki et al., 2012). The authors also elucidated the mechanisms of the reorganizational properties of the NAc, demonstrating that local neural networks from the NAc to the dorsal striatum and cortex showed minimal disruption on day 5 in spared nerve injury (SNI) animals and more extensive reorganization on day 28 post-injury (Baliki et al., 2014). This limbic-cortical connectivity in the SNI model was linked to dopamine receptor gene expression and the degree of tactile allodynia, which together mediated the development of chronic pain (Chang et al., 2014). These findings suggest that the limbic dopaminergic system is involved in the rearrangement of cortical circuits associated with transition to chronic pain.

Further research has revealed that the limbic-cortical functional and anatomical connection not only plays a role in the initial development of pain chronification but can also be a biomarker to predict future disease symptoms in chronic back pain and urologic chronic pelvic pain syndrome (Baliki et al., 2012, Vachon-Presseau et al., 2016). In our study, DMN-BGN connectivity was positively correlated with ID pain scores and was also able to predict the outcome of AHZ patients, with a sensitivity and specificity of 77.8 % and 63.2 %, respectively. This further confirmed the significance of limbic-cortical connectivity in HZ pain chronification and may provide a neuroimaging basis for the early and active treatment of patients with acute HZ and may reduce the incidence of refractory PHN.

Although these are the first findings of network-level connectivity in patients with HZ with different disease courses, the present study had some limitations. First, due to a lack of sufficient follow-up data from patients with acute HZ and the difficulty of a longitudinal study, we simply compared the network connectivity among different populations with different HZ disease courses and did not follow the alterations in the same AHZ population. Future longitudinal studies should emphasize connectivity imbalances within the same acute population. Second, other networks (such as the sensorimotor network, ascending and descending pain modulation pathways) and their within-/cross-network connectivities could also be studied, and we are currently exploring these options. In addition, we could re-run the analyses by using the independent component analysis (ICA) method to verify our results. Finally, as we all know, advanced age is a high risk factor of PHN transition. Although we used age as covariate in our statistical analysis, the significant difference of age among three groups in our study may affect the final predictive function. So in future prospective and longitudinal imaging study, the age would be more consistent among groups to improve on the prediction's effectiveness.

## 5 Conclusion

Overall, the current findings can be placed in a broader context, indicating that brain network imbalance in the acute HZ period, especially limbic-cortical connection, can represent a risk factor for PHN development. This study may provide a neuroimaging basis for the early and active treatment of patients with acute HZ and help to reduce the incidence of refractory PHN.

## Ethical approval

All procedures involving human participants were performed in accordance with the ethical standards of the Ethics Committee of the Second Affiliated Hospital of Zhejiang University, School of Medicine.

## Funding

This work was supported by the National Clinical Key Specialty Construction Project of China (2021-LCZDZK-01) and Zhejiang Medicinal Health, Science and Technology Project (No. 2024KY1063).

## CRediT authorship contribution statement

**Ying Wu:** Writing – review & editing, Methodology, Investigation, Data curation, Conceptualization. **Chao Wang:** Writing – review & editing, Methodology, Formal analysis, Data curation. **Wei Qian:** Formal analysis, Data curation. **Min Yan:** Writing – review & editing, Funding acquisition, Conceptualization. **Lieju Wang:** Writing – review & editing, Methodology, Investigation. **Lina Yu:** Writing – review & editing, Investigation, Conceptualization. **Minming Zhang:** Writing – review & editing, Validation, Conceptualization.

## Declaration of Competing interest

None of the authors have a conflict of interest to declare.

## Data Availability

Data will be made available on request.

## Footnotes

Appendix A Supplementary data associated with this article can be found in the online version at doi:10.1016/j.ibneur.2025.01.009.
