# Supplementary material for: Default mode network-basal ganglia network connectivity predicts the transition to postherpetic neuralgia
Source: IBRO Neurosci Rep. 2025 Jan 13;18:135–41. doi: 10.1016/j.ibneur.2025.01.009 (PMC11783054; doi:10.1016/j.ibneur.2025.01.009)
Supplement: Supplementary file 1 — Supplementary material [file mmc1.docx]

**Supplemental Table 1.** Comparison of within- and cross-network functional connectivities between PHN，RHZ and AHZ patients. (FDR correction).

| FC  (mean ± SD) | PHN | RHZ | AHZ | *p* value  (FDR corrected) |
| --- | --- | --- | --- | --- |
| within-DMN | 0.334 ± 0.329 | 0.381 ± 0.356 | 0.415 ± 0.281 | 0.1095 |
| DMN-SN | -0.171 ± 0.169 | -0.173 ± 0.208 | -0.113 ± 0.162 | 0.0778 |
| DMN-ERN | 0.053 ± 0.213 | 0.052 ± 0.272 | 0.083 ± 0.213 | 0.4618 |
| DMN-BGN | 0.002 ± 0.133 ^c^ | -0.053 ± 0.109 | -0.045 ± 0.117 ^a^ | **0.0135 *** |
| within-SN | 0.418 ± 0.354 | 0.472 ± 0.3 | 0.458 ± 0.288 | 0.7533 |
| SN-ERN | 0.1 ± 0.237 | 0.064 ± 0.297 | 0.158 ± 0.213 | 0.0809 |
| SN-BGN | 0.18 ± 0.147 | 0.202 ± 0.155 | 0.182 ± 0.125 | 0.7526 |
| within-ERN | 0.192 ± 0.306 | 0.166 ± 0.327 | 0.199 ± 0.3 | 0.6412 |
| ERN-BGN | 0.1 ± 0.203 | 0.136 ± 0.233 | 0.127 ± 0.172 | 0.5373 |
| within-BGN | 0.476 ± 0.306 ^c^ | 0.649 ± 0.192 | 0.488 ± 0.331 ^b^ | **0.0174 *** |

* Significant difference between PHN, RHZ and AHZ patients

^a^ Significant difference between AHZ and PHN patients

^b^ Significant difference between AHZ and RHZ patients

^c^ Significant difference between PHN and RHZ patients

PHN, postherpetic neuralgia; RHZ, recuperation from herpes zoster; AHZ, acute herpes zoster; DMN, default mode network; SN, salience network; ERN, emotion regulation network; BGN, basal ganglia network.
