# Supplementary material for: Default mode network-basal ganglia network connectivity predicts the transition to postherpetic neuralgia
Source: IBRO Neurosci Rep. 2025 Jan 13;18:135–41. doi: 10.1016/j.ibneur.2025.01.009 (PMC11783054; doi:10.1016/j.ibneur.2025.01.009)
Supplement: Supplementary file 2 — Supplementary material [file mmc2.docx]

**Supplemental Table 2.** Partial correlation analysis between clinical symptoms and within- and cross-network connectivities in AHZ patients.

|  | **Within**  **-DMN** | **DMN**  **-SN** | **DMN**  **-ERN** | **DMN**  **-BGN** | **Within**  **-SN** | **SN**  **-ERN** | **SN**  **-BGN** | **Within**  **-ERN** | **ERN**  **-BGN** | **Within**  **-BGN** |
| --- | --- | --- | --- | --- | --- | --- | --- | --- | --- | --- |
| **Duration** | 0.373 | 0.017 | 0.143 | -0.027 | -0.146 | -0.075 | 0.188 | 0.072 | 0.139 | 0.127 |
| **MPQ sensory** | -0.094 | -0.075 | -0.024 | 0.006 | -0.251 | -0.308 | -0.332 | -0.171 | -0.232 | -0.131 |
| **MPQ affective** | -0.022 | -0.011 | 0.233 | 0.093 | 0.093 | -0.125 | 0.203 | -0.006 | -0.067 | 0.097 |
| **VAS** | -0.068 | -0.062 | -0.018 | 0.186 | -0.248 | 0.018 | -0.161 | 0.049 | 0.089 | -0.105 |
| **PPI** | 0.11 | -0.104 | -0.083 | 0.187 | 0.039 | 0.08 | -0.003 | 0.163 | 0.021 | 0.131 |
| **ID PAIN** | 0.061 | -0.039 | -0.081 | .432* | 0.044 | -0.032 | -0.071 | 0.14 | 0.207 | -0.022 |
| **HAMD** | -0.043 | -0.031 | 0.328 | 0.108 | 0.083 | -0.015 | 0.133 | 0.189 | 0.182 | 0.135 |
| **HAMA** | 0.21 | -0.171 | 0.349 | 0.045 | 0.144 | -0.2 | 0.021 | -0.103 | -0.121 | -0.123 |
| **PANAS pos** | -0.254 | 0.082 | -0.001 | 0.162 | -0.162 | -0.025 | -0.034 | -0.018 | 0.255 | -0.105 |
| **PANAS neg** | -0.058 | -0.186 | 0.37 | 0.061 | 0.218 | -0.216 | 0.342 | -0.139 | -0.02 | -0.017 |
| **SF-36** | 0.332 | -0.085 | -.409* | -0.175 | -0.172 | 0.182 | -0.184 | 0.057 | 0.192 | 0.003 |

AHZ, acute herpes zoster; VAS, Visual Analogous Scale; MPQ, McGill pain questionnaire; PPI, present pain intensity; HAMD, Hamilton Depression Scale; HAMA, Hamilton Anxiety Scale; PANAS, Positive Affect Negative Affect Score; SF-36, 36-item short form from health survey; DMN, default mode network; SN, salience network; ERN, emotion regulation network; BGN, basal ganglia network.
